# Meta-Analytical Accuracy of ANCA Renal Risk Score for Prediction of Renal Outcome in Patients With ANCA-Associated Glomerulonephritis

**DOI:** 10.3389/fmed.2021.736754

**Published:** 2022-01-06

**Authors:** Mengdi Xia, Ruiran Yu, Zaiqiong Zheng, Huan Li, Jie Feng, Xisheng Xie, Dongming Chen

**Affiliations:** ^1^Nanchong Key Laboratory of Basic Science and Clinical Research on Chronic Kidney Disease, Department of Nephrology, The Second Clinical Medical Institution of North Sichuan Medical College (Nanchong Central Hospital), Nanchong, China; ^2^Department of Oncology, Anqing First People's Hospital of Anhui Medical University, Anqing, China; ^3^Department of Neurosurgery, The First Affiliated Hospital of Anhui University of Traditional Chinese Medicine, Hefei, China

**Keywords:** ANCA-GN, end-stage renal disease (ESRD), renal risk score, meta-analysis, predictive value

## Abstract

**Background:** To evaluate the diagnostic accuracy of antineutrophil cytoplasmic antibody (ANCA) renal risk score (ARRS) for prediction of renal outcome in patients with ANCA-associated glomerulonephritis (ANCA-GN).

**Methods:** We searched PubMed, EMBASE, Ovid, Web of Science, the Cochrane Library, and ClinicalTrials.gov for studies, which used ARRS to predict end-stage renal disease (ESRD) in patients with ANCA-GN. Two reviewers independently screened articles for inclusion, assessed the quality of studies with both an adapted Quality Assessment of Diagnostic Accuracy Studies 2 (QUADAS-2) tool. We calculated the combined patients with ESRD in the ARRS categories and presented the summary and individual estimates based on the ARRS categories. Then, the sensitivity, specificity, diagnostic odds ratio (DOR), positive/negative likelihood ratio, and the area under the receiver operating characteristic (AUROC) curves of the pooled data for ARRS were used to assess the accuracy of the “above the low-risk threshold” (ARRS ≥ 2) and “high-risk grade” (ARRS ≥ 8) for renal outcome of patients with ANCA-GN. The hierarchical summary ROC (HSROC) was used to verify the accuracy value. The clinical utility of ARRS was evaluated by the Fagan plot. Heterogeneity was explored using meta-regression and subgroup analysis.

**Results:** A total of 12 distinct cohorts from 11 articles involving 1,568 patients with ANCA-GN were analyzed. The cumulative patients with ESRD at the maximum follow-up of 60 months was 5% (95% CI: 0.02–0.07; *p* < 0.001) for ANCA-GN with low ARRS (0–1 points) and significantly increased to 22% (95% CI: 0.15–0.29; *p* < 0.001) medium ARRS (2–7 points). The combined cumulative patients with ESRD was 59% (95% CI: 0.49–0.69; *p* < 0.001) high ARRS (8–11 points). The pooled sensitivity of ARRS ≥ 2 in predicting ESRD was 98% with a specificity of 30% and a DOR of 15.08 and the mean AUROC value was 0.82. The pooled sensitivity of ARRS ≥ 8 in predicting ESRD was 58% with a specificity of 86% and a DOR of 7.59. The meta-regression and subgroup analysis indicated that variation in the geographic regions, study design, index risk, follow-up time, age of patient, publication year, and number of patient could be the potential sources of heterogeneity in the diagnosis of ARRS ≥ 8.

**Conclusion:** This meta-analysis emphasized the good performance of the ARRS score in predicting the renal outcome in patients with ANCA-GN. However, these findings should be verified by future large-scale prospective studies.

## Introduction

Antineutrophil cytoplasmic antibody (ANCA)-associated vasculitis (AAV) is a group of life-threatening systemic autoimmune diseases characterized by inflammation, which included granulomatous polyangiitis (GPA), microscopic polyangiitis (MPA), eosinophilic granulomatous polyangiitis (EGPA), and renal-limited vasculitis (RLV) (1). Renal involvement of AAV, which is called ANCA-associated glomerulonephritis (ANCA-GN), occurs in more than 75% of patients with AAV (2, 3), presenting as a pauci-immune necrotizing crescentic ANCA-GN on renal biopsy sample histology and likely to be further developed as end-stage renal disease (ESRD) (4). Renal survival is closely related to prognosis and outcome of patient (5), so identification of the predictive factors for renal survival and outcome in patients with ANCA-GN is very important.

Renal biopsy is a well-established diagnostic modality for the diagnosis of kidney diseases and assessment of activity in ANCA-GN, but the predictive value of renal outcome of ANCA-GN is still controversial (6). In 2010, an international working group of renal pathologists published a histological classification for ANCA-GN based on kidney biopsy. This classification divided patients to four subgroups: focal (>50% normal glomeruli), crescentic (>50% cellular crescents), sclerotic (>50% sclerotic glomeruli), and mixed (any other combination) and the probability of progressing to ESRD increased in ascending order of focal, crescentic, mixed, and sclerotic (7). Some studies have validated that this classification system can reflect the severity of the initial kidney involvement and independently predict the renal outcome (8–10), but lack the influence of clinical factors and interstitial fibrosis (IF) on the prognosis of renal survival.

In the recent years, Brix et al. proposed a validated and predictive tool for ANCA-associated renal vasculitis to estimate the renal survival at baseline, called ANCA renal risk score (ARRS) (11). It is a scoring system that consists of histopathological findings (including the percentage of normal glomeruli, tubular atrophy/interstitial fibrosis) and baseline estimated glomerular filtration rate (eGFR), which ranges from 0 to 11 and three risk groups, from low (0–1 points), medium (2–7 points), and high (8–11 points) probability of ESRD.

The RRS has been validated in several studies among patients with ANCA-GN, though its comprehensive predictive value needs to be confirmed. Therefore, the objective of this systematic review and meta-analysis was to identify and determine the accuracy of ARRS to predict ESRD with patients with ANCA-GN in the baseline.

## Methods

### Search Strategy

The structure of this systematic review conformed to the recommendations from the Preferred Reporting Items for Systematic Reviews and Meta-Analyses (PRISMA) statement (www.prisma-statement.org) (12) and the “Cochrane Handbook for Systematic Reviews of Diagnostic Test Accuracy” was for reviews of diagnostic accuracy (13). The protocol for this systematic review was registered in the International Prospective Register of Systematic Reviews (PROSPERO) database, (Registered No. CRD42021254072).

We performed a systematic search in PubMed, EMBASE, Ovid, Web of Science, the Cochrane Library, and ClinicalTrials.gov from their inception to June 30, 2021. The term was used for (“antineutrophil cytoplasmic antibody” or “ANCA”) and (“ARRS” or “RRS” or “ANCA renal risk score” or “renal risk score”). We did not impose any filter with respect to text availability and there is no restriction placed on language or publication status.

### Eligibility Criteria

The criteria for inclusion of a study in the meta-analysis were as follows: (1) Full-text articles, which investigated the predictive value of ARRS for ESRD in patients with ANCA-GN; (2) original research articles that were written in English; (3) prospective or retrospective studies; and (4) provided sufficient data to calculate the true positive (TP), false positive (FP), true negative (TN), and false negative (FN). We excluded the following studies: (1) meeting abstracts and review articles; (2) case series, case reports, editorials, or letters to the editor that did not include complete data; and (3) lack of adequate information to accurately calculate the test estimates. If there were duplicate publications, we included the most complete version or the article with the highest number of subjects.

### Data Extraction

The included articles will be selected by two independent reviewers (M Xia and D Chen). First, both will review titles and abstracts; second, they will cross-check all the information and disagreements were resolved through consensus. All the extracted data were independently verified by a third investigator (D Chen). From each included study report, we identified the first author, publication year, country, study design, sample size, percentage of male fetuses, follow-up duration, characteristics of included patients (age, histologic class, clinical diagnosis, and antibody subtype), and the number of patients with ANCA-GN who became ESRD. We also extracted data on the index test (including TP, FP, TN, and FN results), accuracy estimates, and data for 2 × 2 tables.

### Quality Assessment

Risk of bias and concerns about applicability was assessed by two authors (M Xia and R Yu) with the Quality Assessment of Diagnostic Accuracy Studies 2 (QUADAS-2) tool (14).

### Data Synthesis

The primary outcome of this systematic review was renal outcome, which we defined as the number of patients with ANCA-GN who became ESRD. The ARRS score was stratified into three categories and we calculated the combined patients with ESRD in the ARRS categories. Summary and individual estimates (proportion of patients with ESRD) were presented graphically with the 95% CIs by a forest plot based on the ARRS categories. We also conducted a diagnostic meta-analysis of the studies that met the criteria and had been screened. Calculate the Spearman's correlation coefficient p between the TP rate and the FP rate and analyze whether there is a threshold effect. If there was no significant threshold effect, the diagnostic accuracy was estimated by pooled statistics. We used summary receiver operating characteristic (SROC) plots to present the results of each study in ROC space, with each study plotted as a single sensitivity specificity point. This produced an SROC curve, with a summary operating point (showing summary sensitivity and specificity values), a summary area under the curve (AUC) value, 95% confidence region, and 95% prediction region. We obtained summary accuracy estimates for the sensitivity, specificity, positive likelihood ratio (PLR), negative likelihood ratio (NLR), diagnostic odds ratio (DOR), and the AUC value and used hierarchical summary ROCs (HSROC) to verify the accuracy value. Using Fagan plot analysis, the post-test probability was calculated under the assumption that the pretest probability was 25, 50, and 75%, respectively. PLR is the ratio of the likelihood of ESRD in those with a positive test vs. those with a negative test. A PLR above 1 indicates increased evidence of ESRD; the farther higher from 1, the more chance of ESRD. NLR is the ratio of the likelihood of ESRD in those with a negative test vs. those with a positive test. NLR below 0.1 is very strong evidence to rule out an ESRD. DOR is the quotient between PLR and NLR. DOR can be calculated as the ratio of the odds of positivity in an ESRD relative to the odds of positivity in the non-ESRD, with higher values, indicating better discriminatory test performance.

### Investigation of Heterogeneity

Heterogeneity among included studies was evaluated using the *I*^2^ and *Q*-statistic and *p* < 0.10 was considered to show significant heterogeneity, *I*^2^ values of 0–40, 40–70, and 70–100% indicate low, moderate, and high heterogeneity, respectively (15). A fixed-effects model was applied when *I*^2^ < 50%, while a random-effects model was applied when *I*^2^ > 50% (16, 17). In the diagnostic meta-analysis, if *I*^2^ > 50% and/or *p* < 0.05 was found, considerable heterogeneity was considered, and in this case, sources of heterogeneity were explored by a subsequent subgroup analysis to identify the potential covariates. Deeks' funnel plot was applied to examine the potential publication bias caused by the asymmetry of the tests. Meta-regression analysis was performed for studies included in the meta-analysis and explored possible sources of heterogeneity (18). We planned to investigate any significant findings on meta-regression using subgroup analyses. Sensitivity analysis was performed to identify the influence of an individual study on pooled estimates by removing one study at a time (17).

### Statistical Analysis

Risk of bias was assessed using the Review Manager version 5.3 (RevMan version 5.3, Copenhagen; The Nordic Cochrane Center, The Cochrane Collaboration, 2014), threshold effect was tested by the Meta-Disc software (version 1.4, Clinical Biostatistics, Ramony Cajal Hospital, Madrid, Spain), and other analysis was conducted on the Stata software (version 14.0, StataCorp, College Station TX).

## Results

### Search Results

**Figure 1:**
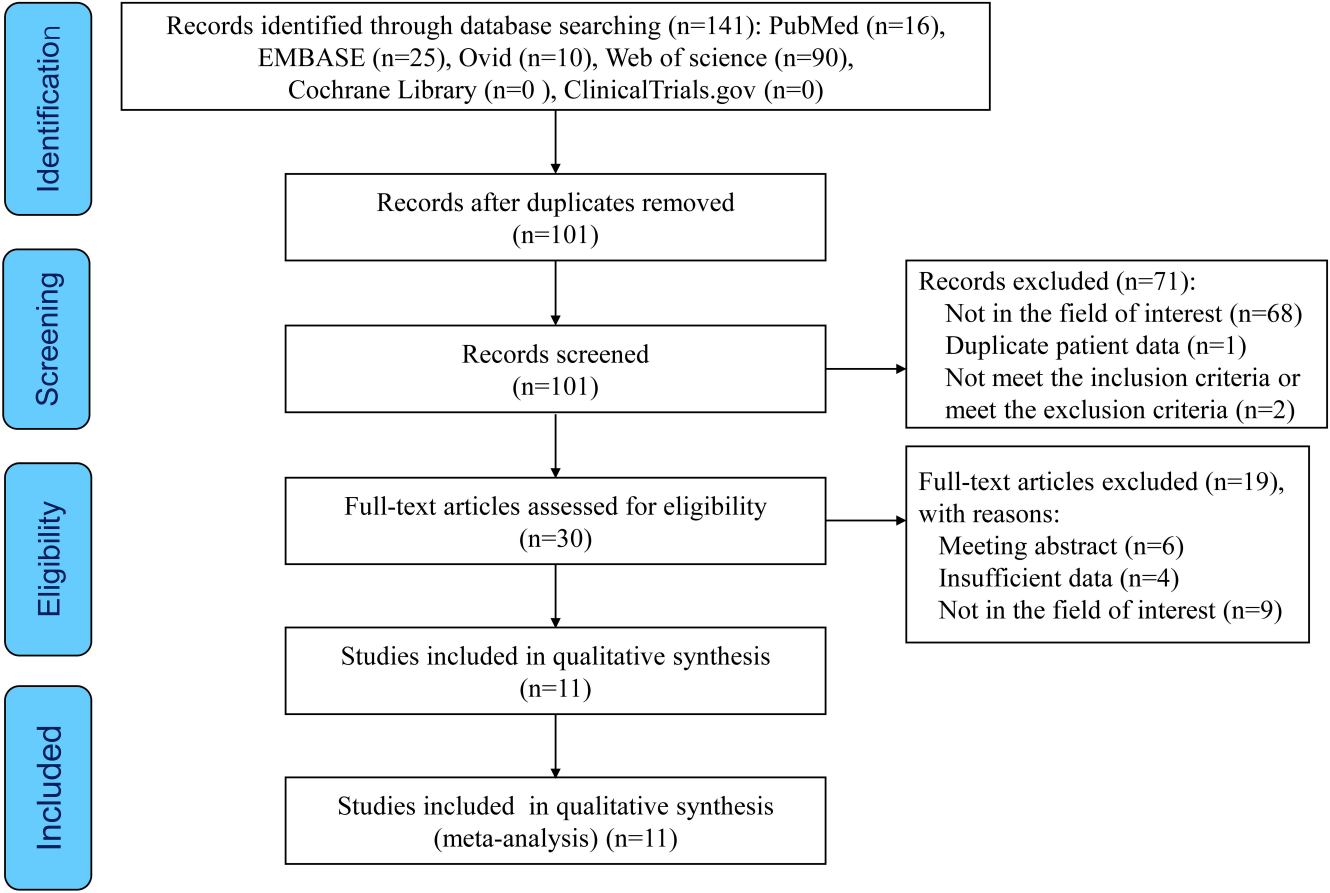
The preferred reporting items for systematic reviews and meta-analyses (PRISMA) flow diagram of this study.

Selection process is given in Figure 1. Of the 141 articles searched, 111 articles were excluded due to duplication (*n* = 40) and irrelevance (*n* = 71) following title and abstract screening. The remaining 30 potentially eligible reports were further evaluated. After excluding the articles with irrelevant contents and articles with no full-text and insufficient data, we included 12 distinct cohorts from 11 articles (11, 19–28) in the meta-analysis.

### Study Characteristics

These 12 studies involving 1,568 patients with ANCA-GN were performed in different geographic regions including Europe (*n* = 6), Asia (*n* = 3), North America (*n* = 1), Africa (*n* = 1), and multicenter (*n* = 1). There was only one prospective cohort and the rest were retrospective cohorts. All the 12 cohort studies of 11 articles evaluated the ARRS score for more than 3 years incidence of ESRD in patients with ANCA-GN. In 12 studies, nine studies described the histologic class of patients. Six studies classified clinical diagnostic subtypes and nine studies detailed antibody subtypes. In these studies, the grading for renal risk was assessed by the ARRS based on the data obtained from baseline estimated glomerular filtration rate (eGFR), the percentage of normal glomeruli, and tubular atrophy/interstitial fibrosis of renal biopsy, as shown in Table 1.

**Table 1:** Base characteristics of included studies.

**Study**	**Region**	**Study design**	**Sample** **(low/** **medium/** **high)** ^†^	**Male (%)**	Age (year)^*^	eGFR (ml/min per 1.73 m^**2**^) ^*^	Follow-up (month)^*^	**Definition of ESRD**	**Histologic class (%)**	**Clinical diagnosis**	**Antibody subtype (%)**
Brix et al. (11) (validation)	Germany	R	90 (26/47/17)	65.6	67.5 (55.3–74)	29.5 (20–44)	31 (20.3–54)	Dialysis or kidney transplantation	F 41.1%, C 27.1%, M/S 37.8%	NR	PR3 (+) 47.8%, MPO (+) 52.2%
Brix et al. (11) (training)	Germany	P	115 (30/64/21)	73	66 (54.5–72)	27.5 (18–47)	34 (22–57)	Dialysis or kidney transplantation	F 33.9%, C 34.8%, M/S 31.3%	NR	PR3 (+) 50.4%, MPO (+) 49.6%
Li et al. (28)	United Kingdom	R	105 (36/51/18)	51.4	66 (57–73)	18 (11–28.5)	42 (26–69)	NR	NR	NR	PR3 (+) 42.9%, MPO (+) 49.5%, Both (-) 7.6%
Gercik et al. (26)	Turkey	R	106 (15/67/24)	57	55 (36–74)	NR	39.6 (24–65)	Permanent dialysis	F 17.0%, C 39.0%, M 31.0%, S 13.0%	MPA 23%, GPA 54%, RLV 18%, EGPA 5%	NR
Jebali et al. (25)	Tunis	R	37 (5/17/15)	48.6	54 (17–80)	16.7 (3–93)	33.15 (1–145)	RRT	F 2.7%, C 24.3%, M 24.3%, S 48.6%	MPA 59.5%, GPA 40.5%	PR3 (+) 40.6%, MPO (+) 59.4%
Daalen et al. (27)	World Wide	R	145 (6/91/48)	NR	63 (55–70)	23 (12–46)	71 (52–126)	NR	F 36%, C 25%, M 27%, S 12%	NR	NR
An et al. (23)	China	R	252 (68/86/98)	44.8	57.5 ± 14.2	20.3 (9.2–45.3)	63.9 ± 49.5	RRT	NR	MPA 84.1%, GPA 4.8%, EGPA 1.6%, RLV 9.5%	MPO (+) 88.1%
Vilet et al. (24)	Mexico	R	72 (13/34/25)	33	53 (35–61)	21 (10–35)	69 (45–98)	RRT or kidney transplantation	F 8%, C 6%, M 35%, S 51%	GPA 56.9%, MPA 25%, RLV 18.1%	PR3 (+) 51%, MPO (+) 25%, Both (+) 6%, Both (-) 18%
Villacorta et al. (21)	Spain	R	147 (32/77/38)	57.8	60.2 ± 16	14.7 (8–27.1)	41 (9.6–104)	Dialysis or renal transplantation	F 19.7%, M 23.8%, C 42.2%, S 14.3%	MPA 38.8%, GPA 6.8%, RLV 54.4%	PR3 (+) 11.6%, MPO (+) 63.9%, Both (+) 0.7%, Both (-) 23.8%
Tan et al. (22)	United Kingdom	R	178 (64/76/38)	NR	NR	NR	NR	NR	NR	NR	NR
You et al. (20)	China	R	70 (12/40/18)	51.4	61.9 ± 10.3	19 ± 8.1	45.9 (20–96)	RRT or kidney transplantation	C 42.9%, M 57.1%	NR	PR3 (+) 5.7%, MPO (+) 88.6%, Both (-) 5.7%
Boudhabhay et al. (19)	French	R	251 (101/76/74)	49.4	63 (52–73)	24 (11–46)	42 (12–89)	Dialysis or kidney transplantation	F 33.9%, C 19.5%, M 23.9%, S 22.7%	RLV 21.1%	PR3 (+) 30.7%, MPO (+) 65.3%, Both (-) 4.4%

*NR, no reported; R, retrospective; P, prospective; RRT, renal replacement therapy; F, focal; C, crescentic; S, sclerotic; M, mixed; GPA, granulomatosis with polyangiitis; MPA, microscopic polyangiitis; EGPA, eosinophilic granulomatosis with polyangiitis; RLV, renal limited vasculitis; PR3, proteinase 3; MPO, myeloperoxidase*.

† *Low denotes ARRS 0–1 points; Medium denotes ARRS 2–7; High denotes ARRS ≥ 8*.

* *Mean ± SD or median (interquartile range)*.

### Quality Assessment

**Figure 2:**
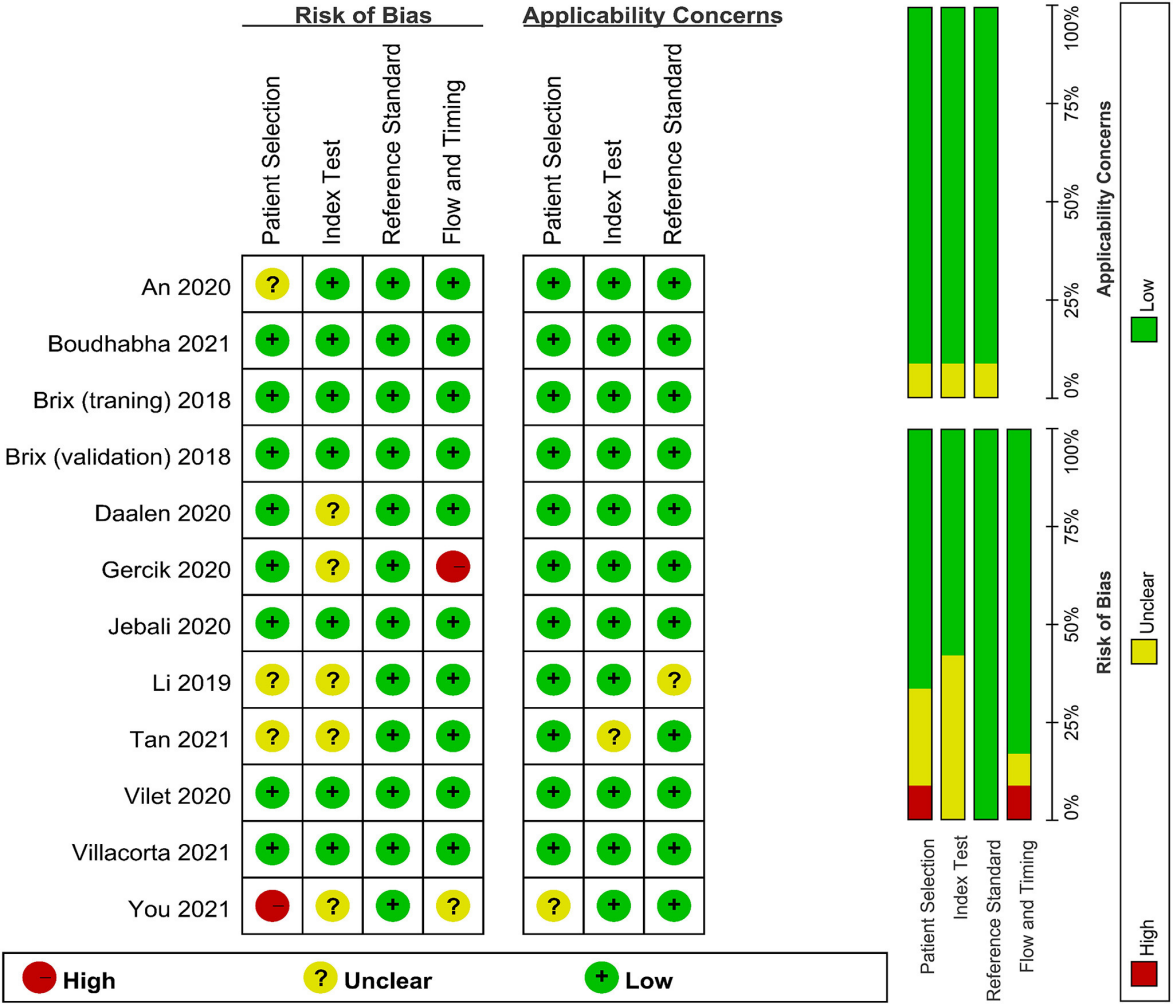
Quality assessment of the included studies.

Quality assessments using the QUADAS-2 criteria are shown in Figure 2. One study was assessed as “high risk” for index test and one study was assessed for flow and timing in the risk of bias. Some studies were estimated as “suboptimal” for unclear risk in the following domains: selection of patient, index test, flow and timing in the risk of bias and selection of patient, index test, and reference standard in the applicability concerns. Most of the studies were identified as having low risk of bias for reference standard in the risk of bias.

### Cumulative ESRD on ARRS Classification

**Figure 3:**
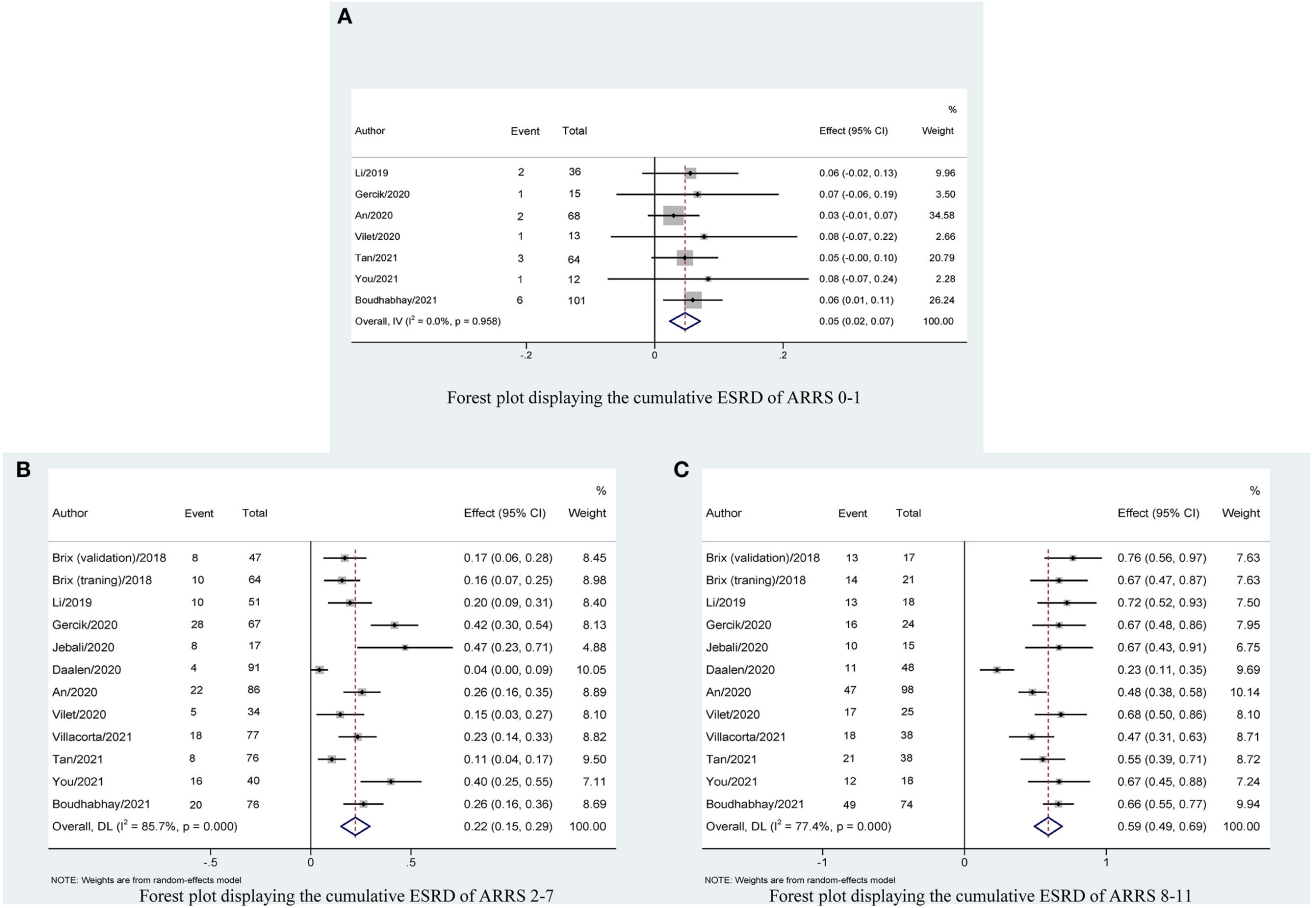
Forest plot displaying the cumulative ESRD with 95% CIs, classified by ARRS scores: **(A)** ARRS scores 0–1; **(B)** ARRS scores 2–7; and **(C)** ARRS scores 8–11. ESRD, end-stage renal disease; ARRS, antineutrophil cytoplasmic antibody renal risk score.

We identified 12 published cohort studies that reported the cumulative patients with ESRD in three ARRS grades. The cumulative patients with ESRD at the maximum follow-up of 60 months was 5% (95% CI: 0.02–0.07; *p* < 0.001) with low heterogeneity (*I*^2^ = 0%, *p* = 0.958) for ANCA-GN with low ARRS (0–1 points) and significantly increased to 22% (95% CI: 0.15–0.29; *p* < 0.001) with high heterogeneity (*I*^2^ = 85.7%, *p* < 0.001) for ANCA-GN with medium ARRS (2–7 points) and the combined cumulative patients with ESRD was 59% (95% CI: 0.49–0.69; *p* < 0.001) with high heterogeneity (*I*^2^ = 77.4%, *p* < 0.001) for ANCA-GN with high ARRS (8–11 points), as shown in Figure 3.

### Overall Predictive Accuracy of ARRS for ESRD

**Figure 4:**
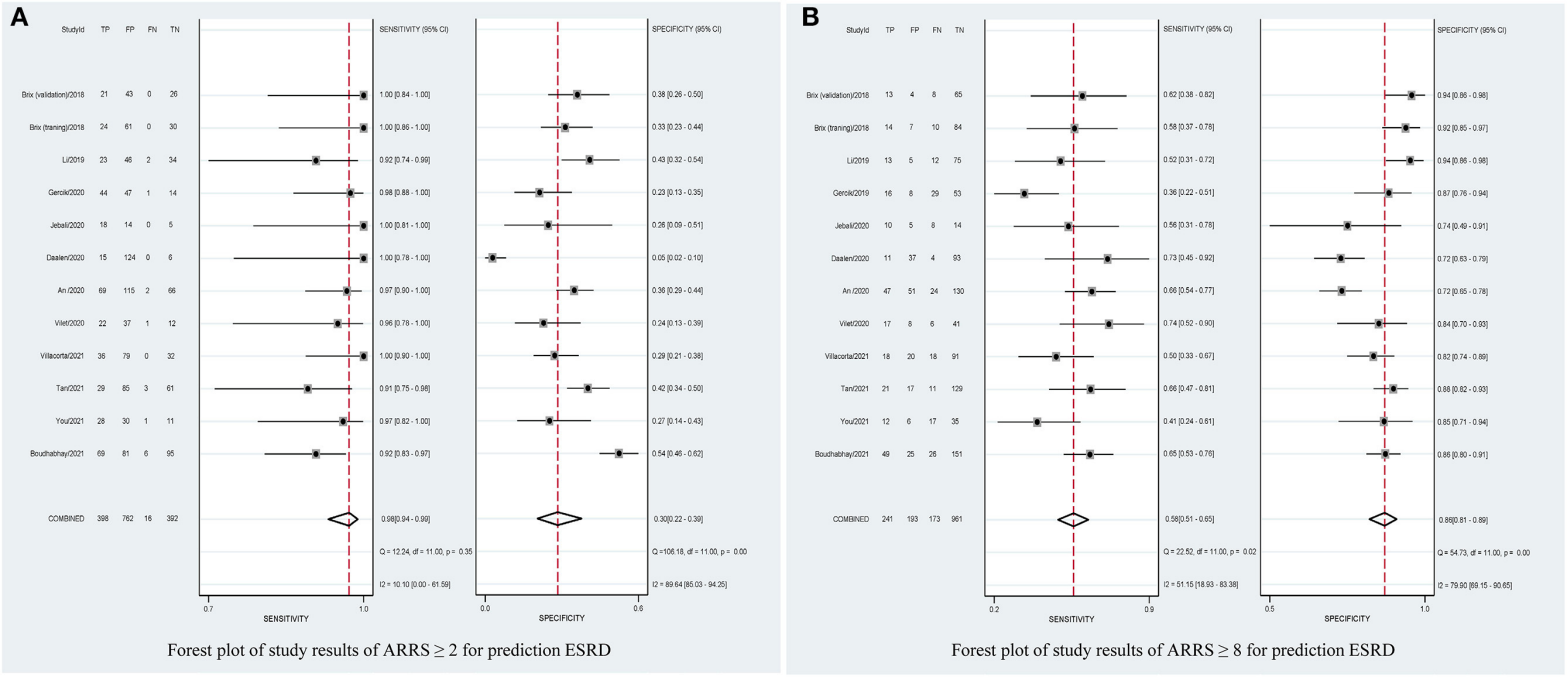
Forest plots of pooled sensitivity and specificity of ARRS ≥ 2 **(A)** and ARRS ≥ 8 and **(B)** for prediction ESRD. ESRD, end-stage renal disease; ARRS, antineutrophil cytoplasmic antibody renal risk score; FN, false negative; TN, true negative; FP, false positive; TP, true positive.

**Figure 5:**
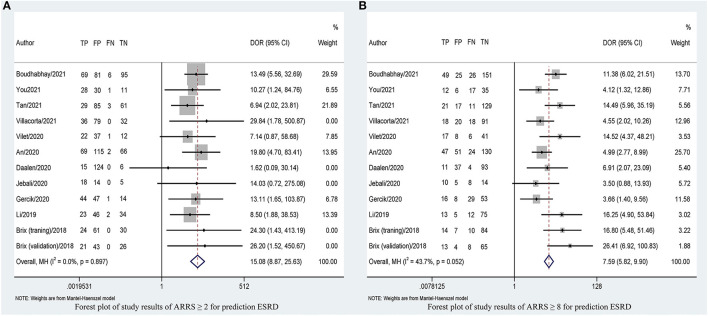
Forest plots of DOR of ARRS ≥ 2 **(A)** and ARRS ≥ 8 and **(B)** for prediction ESRD. ESRD, end-stage renal disease; ARRS, antineutrophil cytoplasmic antibody renal risk score; DOR, diagnostic odds ratio; FN, false negative; TN, true negative; FP, false positive; TP, true positive.

**Figure 6:**
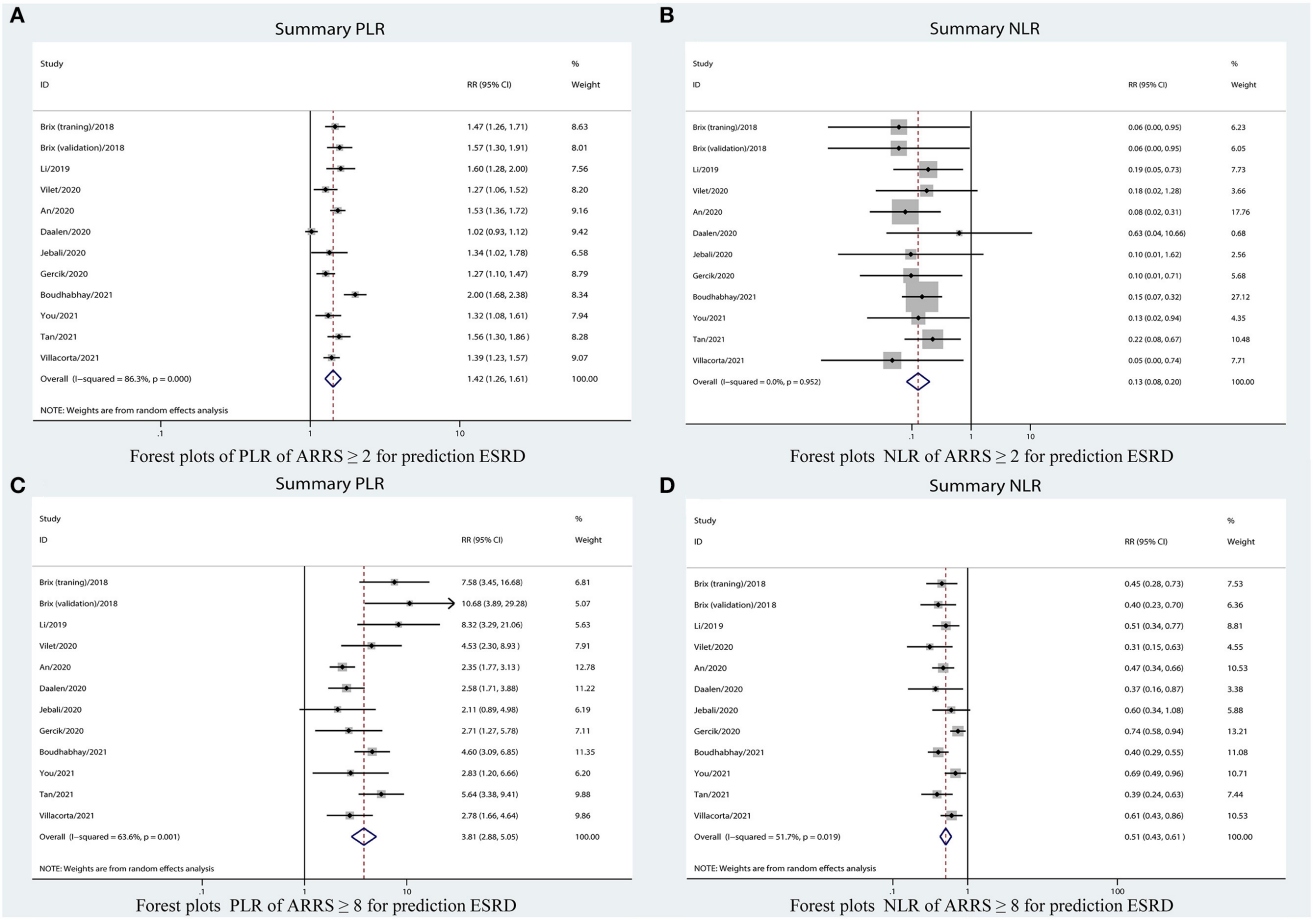
Forest plots of LR of ARRS for prediction ESRD. **(A)** PLR for ARRS ≥ 2; **(B)** NLR for ARRS ≥ 2; **(C)** PLR for ARRS ≥ 8; and **(D)** NLR for ARRS ≥ 8. ESRD, end-stage renal disease; ARRS, antineutrophil cytoplasmic antibody renal risk score; PLR, positive likelihood ratio; NLR, negative likelihood ratio.

**Figure 7:**
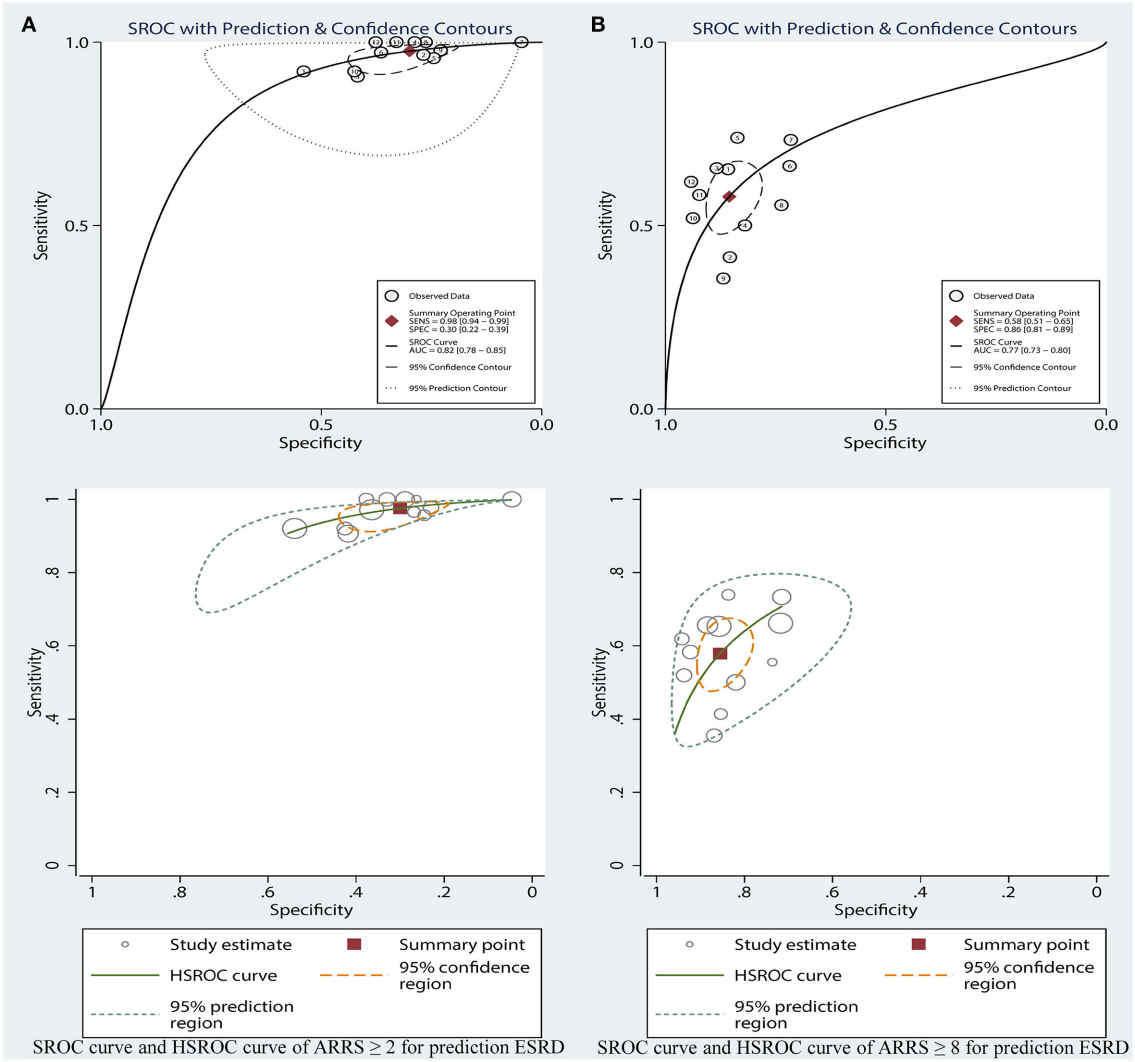
SROC curve and HSROC curve of ARRS ≥ 2 **(A)** and ARRS ≥ 8 and **(B)** for prediction ESRD. ESRD, end-stage renal disease; ARRS, antineutrophil cytoplasmic antibody renal risk score; SROC, summary receiver operating characteristic; HSROC, hierarchical summary receiver operating characteristic.

Table 2 shows that the pooled sensitivity of ARRS score ≥ 2 (above the low-risk threshold) across all the included studies was 0.98 (95% CI: 0.94–0.99, Figure 4A) and specificity was 0.30 (95% CI: 0.22–0.39, Figure 4A). The DOR for positive ESRD was 15.08 (95% CI: 8.87–25.63, Figure 5A), the pooled PLR was 1.42 (95% CI: 1.26–1.61, Figure 6A), the NLR was 0.13 (95% CI: 0.08–0.20, Figure 6B), and the AUC was 0.82 (95% CI: 0.78–0.85, Figure 7A).

**Table 2:** Predictive accuracies of ARRS.

**Category**	**ARRS≥2**			**ARRS≥8**		
	**Data (95% CI)**	***I**^**2**^***(%) or** Z^*^	***p***	**Data (95% CI)**	***I**^**2**^***(%) or** Z^*^	***p***
SE %	0.98 (0.94–0.99)	10.1	0.35	0.58 (0.51–0.65)	51.15	0.020
SP %	0.30 (0.22–0.39)	89.64	<0.001	0.86 (0.81–0.89)	79.9	<0.001
PLR	1.42 (1.26–1.61)	86.3	<0.001	3.81 (2.88–5.05)	63.6	0.001
NLR	0.13 (0.08–0.20)	0	0.952	0.51 (0.43–0.61)	51.7	0.019
DOR	15.08 (8.87–25.63)	0	0.897	7.59 (5.82–9.90)	43.7	0.052
AUROC	0.82 (0.78–0.85)	–	–	0.77 (0.73–0.80)	–	–
Beta^#^	−0.28 (−1.19–0.64)	−0.6	0.552	0.41 (−0.54–1.37)	0.85	0.394
Lambda^#^	2.23 (0.76–3.71)	–	–	1.84 (1.22–2.45)	–	–

*ARRS, ANCA renal risk score; SE, sensitivity; SP, specificity; PLR, positive likelihood ratio; NLR, negative likelihood ratio; DOR, diagnostic odds ratio; AUROC, area under the receiver operating characteristic curve; CI, confidence interval*.

# *From hierarchical summary receiver operating curves (HSROC) model*.

* *Z value only for Beta row, I^2^ value for rest*.

The sensitivity of high-risk ARRS score ≥ 8 for prediction ESRD was 0.58 (95% CI: 0.51–0.65, Figure 4B) and the specificity was 0.86 (95% CI: 0.81–0.89, Figure 4B). The DOR for positive ESRD was 7.59 (95% CI: 5.82–9.90, Figure 5B), the pooled PLR was 3.81 (95% CI: 2.88–5.05, Figure 6C), the NLR was 0.51 (95% CI: 0.43–0.61, Figure 6D), and the AUC was 0.77 (95% CI: 0.73–0.80, Figure 7B). However, these estimates should be interpreted with caution, since considerable heterogeneity was observed in some results.

Corresponding curves from the HSROC model are given in Figure 7. The estimated value of *beta* was 0.41 (95% CI: −0.54 to 1.45), *Z* was 0.85, and *p* > 0.01 for ARRS score ≥ 8, which indicated the symmetrical of SROC curve. 1.84 (95% CI: 1.22–2.45) of lambda verified that the high-risk ARRS score has a high accuracy for predicting ESRD.

### Clinical Utility of ARRS for ESRD

**Figure 8:**
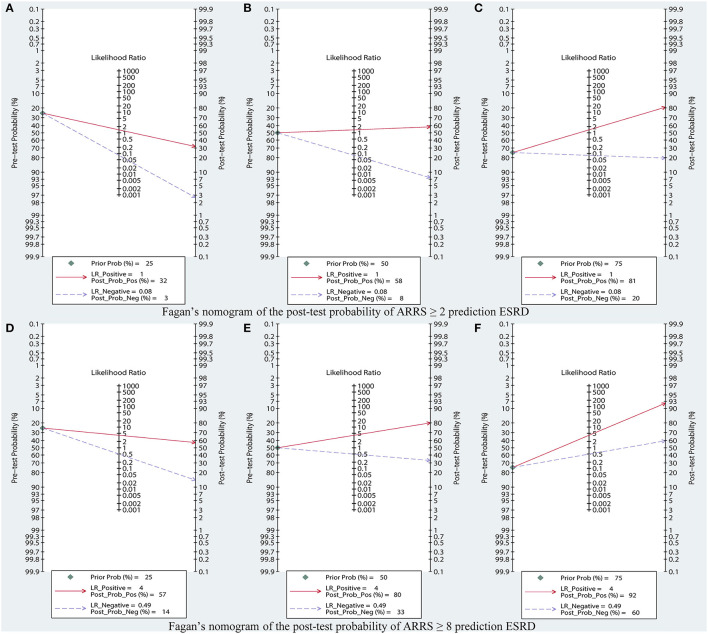
Fagan's nomogram of the post-test probability of ARRS prediction ESRD, based on **(A)** pretest probability = 25%; **(B)** pretest probability = 50%; **(C)** pretest probability = 75% in ARRS ≥ 2; **(D)** pretest probability = 25%; **(E)** pretest probability = 50%; **(F)** pretest probability = 75% in ARRS ≥ 8. ESRD, end-stage renal disease; ARRS, antineutrophil cytoplasmic antibody renal risk score.

In suspected patients with ESRD with score ≥ 2, the Fagan plot analysis revealed the PLR and NLR of 1 and 0.08, respectively. Thus, in this group of patients with 25% pretest probability (based on clinical suspicion), a positive ESRD value revealed a 32% probability of correct diagnosis and a negative ESRD value revealed a 3% probability of wrong diagnosis (Figure 8A). When the pretest probability (based on clinical suspicion) was set to 50%, a positive ESRD value yielded 58% probability of correct diagnosis and a negative ESRD value yielded 8% probability of wrong diagnosis (Figure 8B). When the pretest probability (based on clinical suspicion) was set to 75%, a positive ESRD value showed 81% probability of correct diagnosis and a negative ESRD value showed 20% probability of wrong diagnosis (Figure 8C).

In suspected patients with ESRD with scores 8–11, the Fagan plot analysis revealed the PLR and NLR of 4 and 0.49, respectively. Thus, in this subset of patients with 25% pretest probability (based on clinical suspicion), a positive ESRD value represented a 57% probability of correct diagnosis and a negative ESRD value indicated a 14% probability of wrong diagnosis (Figure 8D). When the pretest probability (based on clinical suspicion) was set to 50%, a positive ESRD value showed 80% probability of correct diagnosis and a negative ESRD value showed 33% probability of wrong diagnosis (Figure 8E). When the pretest probability (based on clinical suspicion) was set to 75%, a positive ESRD value showed 92% probability of correct diagnosis and a negative ESRD value showed 60% probability of wrong diagnosis (Figure 8F).

### Threshold Effect

Calculate the Spearman's correlation coefficient *p* between the sensitivity logarithm and the (1-specificity) logarithm and the *p* of score ≥ 2 and score ≥ 8, the values were 0.389 (*p* = 0.212) and 0.308 (*p* = 0.331), respectively. *p* > 0.05 indicated that there was no threshold effect.

### Meta-Regression and Subgroup Analysis

**Figure 9:**
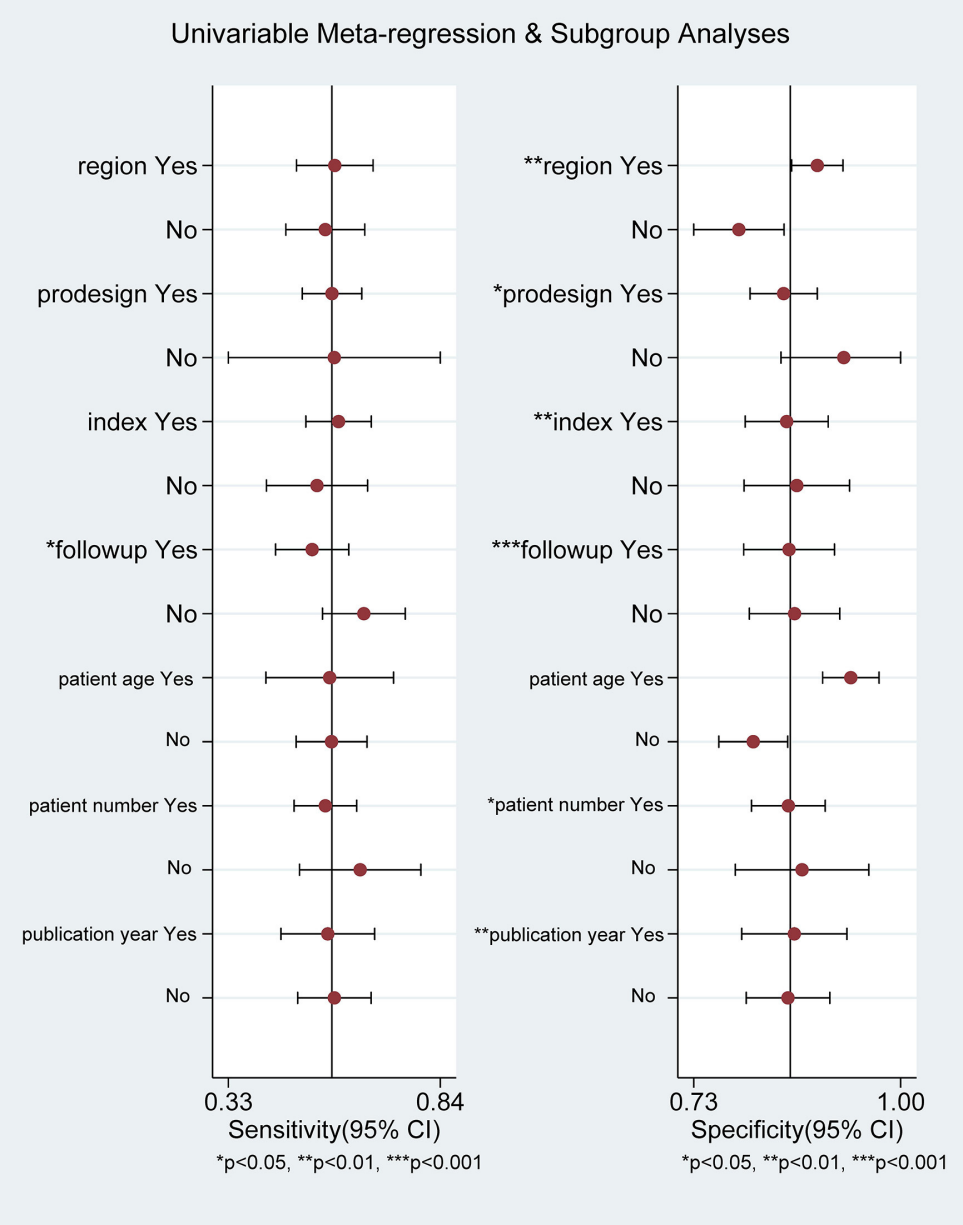
Meta-regression analysis examining heterogeneity.

Overall, result of specificity showed significant heterogeneity; we explored the potential sources of heterogeneity from the region (Europe vs. non-Europe), study design (prospective vs. retrospective), index risk (low risk vs. non-low risk), follow-up (<36 vs. ≥36 months), age of patient (<65 vs. ≥65 years), publication year (<2021 vs. ≥2021), and number of patient (<100 vs. ≥100). The meta-regression analyses showed that all of these values were associated with greater heterogeneity in specificity among ARRS score ≥8 (except for age of patient), as shown in Figure 9.

Table 3 also shows the results of the subgroup analyses. In subgroup analyses, the predictive performance of ARRS was approximately consistent across the multiple subgroups.

**Table 3:** Analysis of subgroups.

**Subgroups**	**Case** **(*n*)**	**ARRS** **≥** **2**				**ARRS** **≥** **8**			
		**SE** **(95% CI)**	**SP** **(95% CI)**	**DOR** **(95% CI)**	**AUROC** **(95% CI)**	**SE** **(95% CI)**	**SP** **(95% CI)**	**DOR** **(95% CI)**	**AUROC** **(95% CI)**
**Region**									
Europe	6	0.96	0.39	12.78	0.71	0.59	0.89	11.79	0.65
		(0.92–0.99)	(0.29–0.50)	(6.97–23.44)	(0.67–0.75)	(0.49–0.68)	(0.86–0.92)	(7.36–18.89)	(0.61–0.69)
Except Europe	6	0.98	0.21	12.12	0.94	0.56	0.79	5.21	0.77
		(0.97–0.99)	(0.13–0.30)	(5.22–28.13)	(0.91–0.96)	(0.47–0.66)	(0.73–0.85)	(3.54–7.69)	(0.73–0.80)
**Study design**									
Retrospective	11	0.97	0.3	12.13	0.83	0.58	0.85	7.29	0.77
		(0.94–0.99)	(0.21–0.40)	(7.36–20.01)	(0.79–0.86)	(0.51–0.65)	(0.80–0.89)	(5.55–9.58)	(0.73–0.81)
Prospective	1	–	–	–	–	–	–	–	–
**Index**									
Yes	8	0.98	0.33	15.9	0.78	0.59	0.85	7.39	0.7
		(0.96–0.99)	(0.23–0.44)	(8.59–29.41)	(0.74–0.81)	(0.52–0.67)	(0.80–0.91)	(5.43–10.07)	(0.66–0.74)
No	4	0.97	0.24	7.52	0.75	0.54	0.86	8.19	0.8
		(0.93–1.00)	(0.12–0.36)	(3.28–17.26)	(0.71–0.79)	(0.42–0.66)	(0.79–0.93)	(4.88–13.75)	(0.76–0.83)
**Follow-up**									
≤ 36 months	6	0.97	0.35	9.65	0.71	0.66	0.86	11.57	0.73
		(0.94–1.00)	(0.23–0.48)	(4.13–22.45)	(0.67–0.75)	(0.56–0.76)	(0.80–0.92)	(7.22–18.55)	(0.69–0.76)
>36 months	6	0.98	0.25	14.35	0.81	0.53	0.85	6.25	0.77
		(0.96–0.99)	(0.15–0.36)	(7.85–26.25)	(0.78–0.84)	(0.44–0.62)	(0.79–0.91)	(4.52–8.63)	(0.73–0.80)
**Patients age**									
≥65	3	0.97	0.37	12.6	–	0.57	0.93	18.78	–
		(0.93–1.00)	(0.19–0.56)	(3.77–42.14)	–	(0.42–0.73)	(0.90–0.97)	(9.34–37.75)	–
<65	8	0.98	0.26	11.32	0.83	0.58	0.81	6.06	0.78
		(0.96–1.00)	(0.16–0.36)	(6.61–19.38)	(0.80–0.86)	(0.49–0.66)	(0.76–0.85)	(4.23–8.67)	(0.75–0.82)
**Patient number**									
≥100	9	0.97	0.3	11.47	0.81	0.56	0.85	7.56	0.77
		(0.95–1.00)	(0.20–0.40)	(6.81–19.32)	(0.78–0.84)	(0.49–0.64)	(0.80–0.90)	(5.09–11.21)	(0.73–0.80)
<100	3	0.99	0.29	11.92	–	0.65	0.87	11.25	–
		(0.95–1.00)	(0.11–0.47)	(2.74–51.95)	–	(0.50–0.79)	(0.78–0.96)	(3.64–34.71)	–
**Publication year**									
≥2021	4	0.96	0.38	11.33	0.81	0.57	0.86	7.92	0.85
		(0.91–1.00)	(0.23–0.53)	(5.84–21.96)	(0.77–0.84)	(0.46–0.68)	(0.79–0.93)	(4.35–14.39)	(0.81–0.88)
<2021	8	0.98	0.26	11.76	0.88	0.58	0.85	8.43	0.76
		(0.96–1.00)	(0.17–0.36)	(5.65–24.49)	(0.85–0.91)	(0.50–0.67)	(0.80–0.91)	(4.99–14.22)	(0.72–0.79)

*AUROC, area under the receiver operating characteristic curve; SE, sensitivity; SP, specificity; DOR, diagnostic odds ratio; ARRS, ANCA renal risk score; Index: Index text is low risk*.

### Sensitivity Analysis

The sensitivity analysis was performed by reducing one article each time to evaluate the impact of a single study on this meta-analysis. Sensitivity analyses for the proportion of patients with ESRD and the result showed that removing single studies that did not have any significant impact on the final outcome. The combined DOR after each elimination had not changed significantly, showing that the results of this analysis are not excessively dependent on a certain study and the conclusion is stable.

### Publication Bias

**Figure 10:**
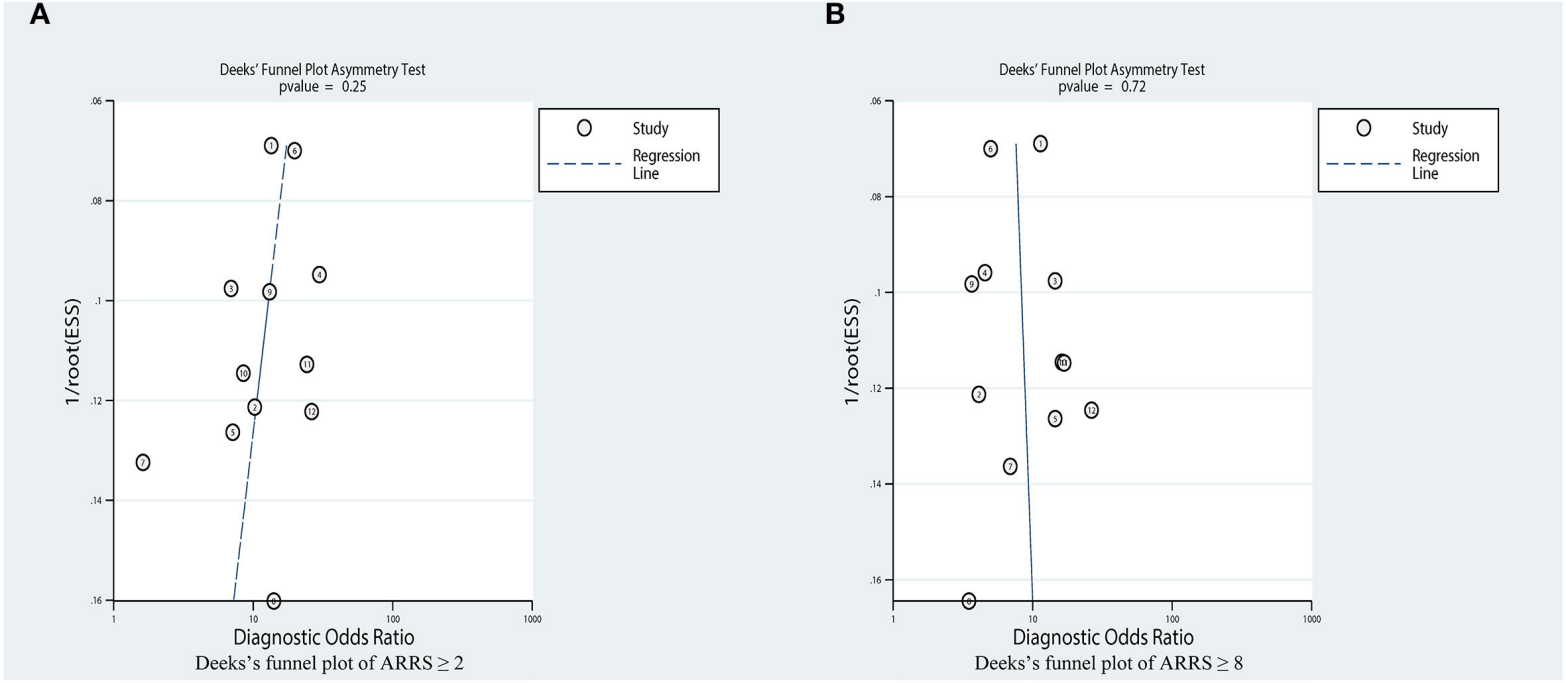
Estimation of the publication bias by Deeks' funnel plots. **(A)** Analysis on the publications concerning ARRS ≥ 2 and **(B)** Analysis on the publications concerning ARRS ≥ 8. ARRS, antineutrophil cytoplasmic antibody renal risk score; ESS, effective sample size.

Based on the Deeks' Funnel plot, publication bias was also not detected in the studies where ESRD was used to detect ARRS score ≥ 2 (*p* = 0.25, Figure 10A) and ARRS score ≥ 8 (*p* = 0.78, Figure 10B).

## Discussion

Associated vasculitis has a high proportion of kidney injury (29). Despite current therapy improving the prognosis of patients, ANCA-GN still results in a rapid or gradual deterioration in renal function (30). ESRD caused by kidney involvement is an important adverse prognostic factor and is associated with high mortality (1, 31). In the past few decades, scholars have tried to specify the characteristics of renal involvement to determine the factors that affect the prognosis of the kidney (32, 33) and have recently developed an ARRS scoring system for predicting renal outcome.

To the best of our knowledge, this is the first systematic review and meta-analysis to report the renal outcome prediction in patients with ANCA-GN using ARRS. Our results confirmed that the pooled incidence rate of ESRD was 4, 22, and 58% in the low-, medium-, and high-risk groups, respectively. It means that patients with the higher ARRS score had a higher probability of suffering ESRD in the next 3–5 years and who had low-risk grade (ARRS score of 0) should be informed about the low possibility of suffering ESRD; this was consistent with previous validation studies. In the meta-analysis of diagnostic tests part, the diagnostic performance of ARRS in different risk grades of ESRD was evaluated in patients with ANCA-GN. Our data revealed that the AUROC of ARRS exhibited a fair diagnostic value for predicting ESRD in ARRS both for ≥ 2 and ≥ 8. Through pooled sensitivity and specificity, it was revealed that ARRS ≥ 2 had a high sensitivity of identifying the potential ESRD and ARRS ≥ 8 had a high accurate predictability of ESRD in patients with ANCA-GN. Fagan plot analysis showed that ARRS ≥ 2 had a low negative LR and wrong diagnosis rate when predicting ESRD, thus reflecting that when ARRS < 2, few developed ESRD, laterally reflecting the good performance of ARRS < 2 (low-risk class) in detecting renal survival. This analysis also set the good predictive value of ARRS ≥ 8 (high-risk grade) for ESRD.

The present scoring system has an advantage of high sensitivity to potential patients with ESRD above the low-risk threshold and high positive predictive value in the high-risk grade of ESRD and these advantages were thought to come from the characteristics of the composition. Kidney biopsy, the “gold standard” in the diagnosis of kidney disease, was reported as a predictor of kidney prognosis in patients with ANCA-GN in 1999. Bajema et al. (34) performed an observational cohort study in biopsies from 157 patients with clinically and histologically confirmed ANCA-GN. Results indicated that the proportion of normal glomeruli in initial renal biopsy is an excellent predictor of renal function in patients with ANCA-GN. They also proposed that the only active lesion that predicts renal function is from the interstitium. The number of diffuse interstitial infiltrates correlated with serum creatinine values at enrollment and during follow-up. Subsequently. Berden et al. (7) proposed a classical histopathological classification based on renal biopsy. They studied 100 biopsies with ANCA-GN, lesions were classified according to their predominant glomerular state, and diagnosed from March, 1995 to September, 2002. This validation study showed focal type, with more than 50% of common glomeruli, has relatively the most intact renal function with better renal outcome. The sclerosing type, with more than 50% sclerosing glomeruli, has a high risk of irreversible severe renal impairment and death at the 1-year follow-up period. In the meantime, the predictive role of the renal interstitium for renal outcome has been repeatedly mentioned. Studies have revealed that the presence of diffuse interstitial fibrosis and high tubular atrophy predicted impaired renal function for patients with ANCA-GN during follow-up and tubular atrophy was important predictors of recovery of renal function and renal outcome, independent of initial renal function (35, 36). The third component of ARRS is the initial eGFR. A European multicenter prospective study confirmed that patients with ANCA-GN with GFR < 50 ml/min/1.73 m^2^ had a 50% chance of developing ESRD (37) and other data from China also suggested that patients with ANCA-GN with serum creatinine (SCr) levels ≥ 4 mg/dl have a nearly three-fold increased risk of ESRD (38). In addition, severe insufficiency of baseline renal function affects the outcome of subsequent AAV treatment. It has been established that patients with severe renal dysfunction were less likely to respond to treatment, have an increased risk of adverse immunotherapy reactions, and an increased risk of ESRD compared to those with preserved renal function (39).

In the diagnostic meta-analysis section, we observed that the heterogeneity between studies was more pronounced in pooled specificity. Meta-regression analyses indicated that region, study design, index risk, age of patient, publication year, number of patient, and subject risk could be the source of heterogeneity. First of all, the ARRS scoring system was proposed in the recent years and the studies validated so far were more scattered and influenced by multiple variables such as geography and population. It can be found in subgroup analysis that heterogeneity can be reduced, but not completely eliminated when analyzed by a single variable. Furthermore, the composition of the scoring system itself may affect the results. It is known that renal outcomes are influenced not only by pathology and initial renal function, but also by multiple factors such as age, genetics, baseline proteinuria, ANCA serology, ANCA antibody subtypes, and treatment (6, 40–43); although some studies have described a possible correlation between these factors and renal pathology histology and baseline renal function (33, 44, 45), the potential influence of these factors may also account for heterogeneity in including studies.

## Limitations and Future Directions

Several limitations of this study should be noted. First, there was significant heterogeneity in the pooled specificity, which may affect its accuracy. We were able to identify some sources of heterogeneity through subgroup analysis and meta-regression analysis. Second, we were unable to adequately assess the associations between subgroups and other factors due to inadequate descriptions of some of the included studies. Third, the limited sample size of the included studies may also affect data interpretation. Therefore, the generalizability of our findings needs further confirmation. Fourth, several included studies were not provided the description of remission induction and maintenance regimens. We, therefore, did not explore the potential influence of remission induction and maintenance regimens on the outcomes of ESRD; however, future studies should explore this issue.

## Conclusion

In summary, this study emphasized the merits of ARRS having a predictive ability for ESRD events in ANCA-GN. This new grading system is associated with moderate to good diagnostic value for predicting ESRD in ANCA-GN and also predicts ESRD rates in different risk patients. Future large-scale prospective studies should be conducted to verify the accurate assessment of the diagnostic value of ARRS for predicting ESRD events that could result in screening high-risk individuals for preparing the renal replacement therapy in advance and assessing the survival prognosis.

## Data Availability Statement

The original contributions presented in the study are included in the article/supplementary material, further inquiries can be directed to the corresponding authors.

## Author Contributions

MX, RY, and DC developed the study question. MX and DC wrote the first draft of the manuscript. DC and XX critically read and revised the final version of the manuscript before submission. All the authors contributed to the development of the review protocol, search strategies, refining of the manuscript, and approved the final manuscript.

## Funding

This study was supported by grant from the Nanchong School Science and Technology Strategic Cooperation Project (20SXQT0117) and the Sichuan Traditional Chinese Medicine Research Project (2020LC0146).

## Conflict of Interest

The authors declare that the research was conducted in the absence of any commercial or financial relationships that could be construed as a potential conflict of interest.

## Publisher's Note

All claims expressed in this article are solely those of the authors and do not necessarily represent those of their affiliated organizations, or those of the publisher, the editors and the reviewers. Any product that may be evaluated in this article, or claim that may be made by its manufacturer, is not guaranteed or endorsed by the publisher.
